# Application Description and Policy Model in Collaborative Environment for Sharing of Information on Epidemiological and Clinical Research Data Sets

**DOI:** 10.1371/journal.pone.0009314

**Published:** 2010-02-19

**Authors:** Elias César Araujo de Carvalho, Adelia Portero Batilana, Julie Simkins, Henrique Martins, Jatin Shah, Dimple Rajgor, Anand Shah, Scott Rockart, Ricardo Pietrobon

**Affiliations:** 1 Research on Research Group, Duke University Health System, Durham, North Carolina, United States of America; 2 Duke-National University of Singapore Graduate Medical School, Singapore, Singapore; 3 Fuqua School of Business, Durham, North Carolina, United States of America; 4 Department of Surgery, Duke University Health System, Durham, North Carolina, United States of America; University of Oxford, United Kingdom

## Abstract

**Background:**

Sharing of epidemiological and clinical data sets among researchers is poor at best, in detriment of science and community at large. The purpose of this paper is therefore to (1) describe a novel Web application designed to share information on study data sets focusing on epidemiological clinical research in a collaborative environment and (2) create a policy model placing this collaborative environment into the current scientific social context.

**Methodology:**

The Database of Databases application was developed based on feedback from epidemiologists and clinical researchers requiring a Web-based platform that would allow for sharing of information about epidemiological and clinical study data sets in a collaborative environment. This platform should ensure that researchers can modify the information. A Model-based predictions of number of publications and funding resulting from combinations of different policy implementation strategies (for metadata and data sharing) were generated using System Dynamics modeling.

**Principal Findings:**

The application allows researchers to easily upload information about clinical study data sets, which is searchable and modifiable by other users in a wiki environment. All modifications are filtered by the database principal investigator in order to maintain quality control. The application has been extensively tested and currently contains 130 clinical study data sets from the United States, Australia, China and Singapore. Model results indicated that any policy implementation would be better than the current strategy, that metadata sharing is better than data-sharing, and that combined policies achieve the best results in terms of publications.

**Conclusions:**

Based on our empirical observations and resulting model, the social network environment surrounding the application can assist epidemiologists and clinical researchers contribute and search for metadata in a collaborative environment, thus potentially facilitating collaboration efforts among research communities distributed around the globe.

## Introduction

Although millions of dollars are spent on the creation and management of biomedical and epidemiological research data sets every year [1], the information extracted from these data sets to improve healthcare and prevention is poor at best, with pathetic perhaps being a better descriptive word. In a system that emphasizes competition rather than collaboration among researchers, data sets resulting from multi-million investments from tax payers sit idle inside locked computers, only available to a small number of researchers despite their containing the seeds that would allow for the exploration of a vast number of important research questions that could change the healthcare landscape. One of the reasons for this lack of sharing is that researchers consider their data proprietary [2], providing them with a competitive advantage over other groups in terms of discovery and further acquisition of funds that would expand their research operations. This unintended consequence of the current organization behind biomedical research ultimately leads to duplication of research efforts, also precluding an expedited path toward the discovery of answers to important research questions.

Previous efforts attempting to address this problem have mostly emphasized the exchange of data, including previous policies developed through NIH [3], as well as systems developed by caBIG [4] and Science Commons [5]. Focusing on data, however, has a downside since most researchers are not willing to share their data for reasons related to the logistical, technical, legal, and ethical aspects of data sharing [6]. Since data sharing is largely driven by a network effect, i.e., one will only share if others also decide to share, these concerns tend to lead individuals and organizations towards hoarding rather than sharing data. Additionally, since sharing involves recurring costs and a sophisticated aparatus to ensure security that are frequently not covered by funding agencies, researchers do not find value in investing in data sharing.

In this context, “metadata” or “information describing the data” becomes an interesting alternative to the sharing of data[7]. metadata provides an overview of what is housed in the database [8] and can be used to facilitate research collaboration, even when the actual data cannot be made publicly available. By promoting awareness among a broad research audience regarding pre-existing data, metadata sharing can prove potentially beneficial to users whose subject interests cross disciplinary boundaries. Of importance, metadata sharing does not lead to researchers losing their ownership and competitive advantage over the data. As an added benefit, sharing metadata represents a publicity tool: Peers will learn about available resources and then approach researchers for future collaborations.

Although the idea of metadata sharing is appealing, it still represents an additional cost, specially when considering long-term costs of maintenance and quality improvement. In an ideal situation, researchers would be able to share metadata in a pre-existing social network, with minimal effort and cost, and maximum exposure to others. Social networks would also have the added benefit of providing researchers with feedback on how useful or how good the quality of their data might be among their collaborators. The literature contains several examples where social networks have been used to improve the quality of information, including success stories such as Wikipedia and other common metadata repositories receiving contributions from multiple users [9]. These contributions are consistently archived, thus describing the history of the metadata and serving as a versioning mechanism that allows researchers to determine whether the metadata is outdated or whether it is still valid. Despite the previous stories of success, to our knowledge no previous social network applications have been used in the sharing of research metadata

In our paper we present one potential approach that, rather than assuming a complete cultural change towards the sharing of data, explores the possibility of sharing of metadata in a social network environment. This approach is demonstrated through the creation of a novel Web application that allows for the sharing of metadata, followed by a policy model that explains how this application fits into a broader social science picture based on feedback from active clinical researchers using the described application.

## Methods

Our project is constituted by two major sections. First we describe the construction and deployment of a Web application to collect and share information on existing biomedical databases. This information is shared among participants with common research interests, ultimately leading to an increase in the sharing of databases toward common publications and collaborative funding proposal. Second, in order to evaluate the underlying mechanism behind this application, we develop a dynamic model based on researcher expert opinion. The model delivers information on the expected mechanisms leading to increased collaboration among researchers.

### Application Design and Development

The Database of Databases (DoD) was developed to serve as a repository of information about epidemiological and clinical research studies. Before developing this application, we analyzed similar tools, including commercially available products, to ensure that there was no duplication of previously available software. Software requirements were defined based on requirements stipulated by epidemiologists and clinical researchers working with our development group as well as review of the literature. Requirements included: simple search interface in addition to an advanced search using pre-specified filters, ability by end users to create information on new databases, a wiki-like mechanism to change information about databases as needed, and an export function of data dictionaries in a spreadsheet format. Also, the technology should be user-friendly so that users can execute these operations and enter data directly through the interface, without requiring assistance from professional programmers. Details about the software architecture, hardware requirements and the DoD workflow are provided in Appendix S1 and figures S1, S2, S3, S4, S5.

### Modeling Strategy

Our modeling strategy was designed to evaluate how different policy strategies would affect the ability and willingness of epidemiologists and clinical researchers to collaborate. Since the opinion of epidemiologists and clinical researchers had to be incorporated based on pre-existing experiences with collaboration models, we selected researchers who had been previously exposed to different incentives to collaborate. We created a policy model using System Dynamics [10], [11] to evaluate the comparative impact of data sharing vs. metadata sharing policies against baseline. System Dynamics has been extensively used in the Research and Development literature as well as healthcare [12]–[20], although evaluate biomedical research processes.

We built three policy models: (1) A baseline model depicting the currently prevalent model that stimulates competition and provides little incentive for collaboration, (2) a data sharing model that provides incentives for collaboration based on requirements from funding agencies similar to recent policies by National institutes of health (NIH), and (3) a metadata sharing model with incentives for collaboration through the sharing of variable level information about databases but not necessarily actual data itself. Input for each of the models came from two sources. First, qualitative interviews with epidemiologists and clinical researchers and research policy experts provided data for the overall model structure, stocks, and causal elements for all three models. Second the policy model was based on information obtained from a focus group and email-based Delphi study involving epidemiologists and clinical researchers and research policy experts. The combination of focus groups and associated Delphi study resulted in an agreement regarding overall model structure, element values as well as information available from the literature whenever possible. All researchers and research policy specialists participating in the focus group were aware and currently using the DoD, also being familiar with institutional and funding agency policies regarding data sharing.

## Results

### General Application Features

The current application contains information on over 130 epidemiological and clinical research databases, with unrestricted search not requiring registration (http://tools.researchonresearch.org/dodsg/). To upload information on new study data sets, users are required to register so that they can then act as mediators for any modification made by contributors to the information on their databases.

### Collaboration Features

To facilitate collaboration, the application allows for user-driven modification similar to collaborative environments such as Wikipedia (www.wikipedia.org). Users can therefore modify the content of the general information about each study database as well as the data dictionary. All changes made through the wiki mechanism are sent to the person initially uploading the database information, for approval before they are publicly displayed, thus creating a protection against spam. For example, Dr. Smith can update his experiences about the quality of a specific variable in Dr. Wong's database. It will be up to Dr. Wong to approve the inclusion of the information for public display, thus creating a balance between crowd intelligence and owner oversight.

Those uploading information about a study database can solicit collaborations by adding a list of research questions that, in their opinion, could be successfully explored. For example, Dr Wong can list a series of research questions to her database page. Graduate students, junior investigators, and other researchers can then take her comments and decide to explore the question in collaborative research project. Alternatively, these researchers can also suggest research questions to Dr. Wong.

Finally, the application is connected to BIOS-Sg (https://tools.researchonresearch.org/ biossg), a biosketch management application that stores information about research interests and publications of a large group of researchers. This connection matches key words from publications of individual researchers with key words describing specific study databases, thus notifying researchers whenever information on new study databases are uploaded into the DoD application. For example, if Dr. Smith has a biosketch in BIOS-Sg, her list of publications generates a list of keywords that is then matched to all information on study databases that might be of interest to her.

### Current Utilization in Collaborative Environments

The DoD currently houses information on databases from researchers and institutions in the US, Australia, China and Singapore, having contributed to a number of publications created by authors in North and South America and Asia (http://www.researchonresearch.org/ ?q = node/2). Its intuitive interface for searching and storing metadata optimizes workflow, and a variety of educational videos assists first-time users familiarize themselves with its interface.

### Results from Focus Group and E-mail Based Delphi Method: Input Data for Model

**Figure 1:**
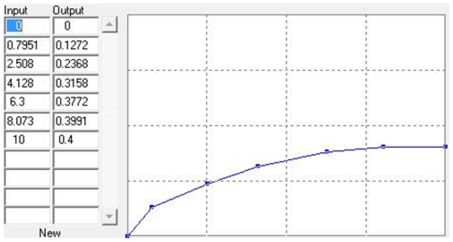
Overall behavior expected by focus group members regarding the impact of publications on ability to obtain further funding for study database.

The following assumptions were extracted from the focus group and e-mail based Delphi method to populate our policy model. The baseline policy model was assumed by the group to describe the competitive nature of biomedical research, with researchers not having incentives to share data since that would make them lose competitive advantage. The data sharing policy model was based on the assumption that a funding agency would enforce the sharing of data. This model was believed to lead to the unintended consequence of a percentage of researchers not appropriately sharing data (data missharing). It was assumed that the misshared data would result in no publications and also in wasted time for researchers attempting to use it. Finally, the metadata sharing policy model provided incentives to sharing of metadata, which then affected the sharing of data. Successful publications were associated with additional funding that would then feed back to the creation of additional databases. Model parameters defined by the focus group included average yearly grant size for a typical study registry ($50,000 to $500,000), impact of publications on ability to obtaining further funding for the study database or subsequent study databases (Figure 1), expected rate of mishared databases (2% to 30%), expected rate of sharing for metadata (90%), funding rate for study proposals (5% to 35%) (http://www.aecom.yu.edu/ogs/NIHInfo/paylines.htm), publishing rate per study database (1 to 5 publications/year). Since these factors were mostly undocumented in the literature, our model aimed for reproducing the general behavior rather than achieving numeric predictions. A sensitivity analysis in model parameters was performed to test behavior under extreme values and ensure its consistency.

**Figure 2:**
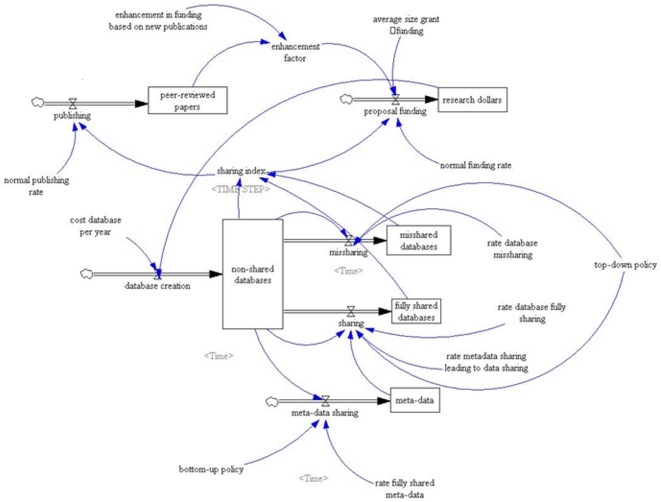
System Dynamics model.

The full model is displayed on Figure 2.

### Policy Model Results

**Figure 3:**
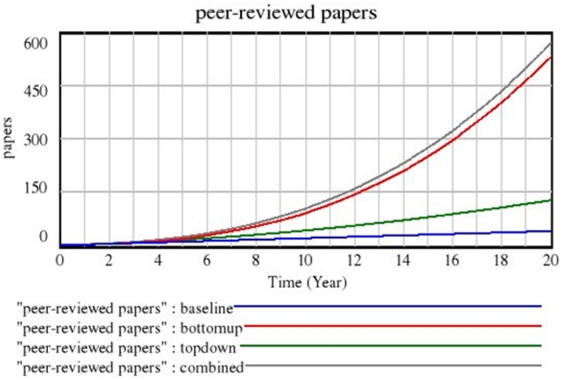
Comparison in number of peer-reviewed papers.

The policy model results can be divided into three types of comparison (Figure 3): (1) Current strategy (baseline) versus any policy, i.e., data-sharing (top-down) or metadata sharing (bottom-up), (2) isolated policies compared (i.e., data-sharing versus metadata sharing) and (3) combined versus isolated policies (i.e., both policies implemented at the same time versus either one implemented in isolation). When comparing the current strategy vs. either the isolated data-sharing or metadata sharing policy, the implementation of any policy consistently led to an improvement in the number of shared databases, ultimately resulting in an increased number of publications per study data set as well as further funding acquisition from all sources. While the current strategy tended to lead to a steady, linear growth, all other strategies lead to an exponential growth which is explained by the growing number of publications and funding obtained by sharing.

When comparing the introduction of isolated data-sharing versus metadata sharing policies, a metadata sharing policy resulted in a greater number of shared databases, resulting publications, and funding. This finding can be attributed to the unintended consequence of mishared data sets associated with the data-sharing policy, which introduced more wasted time in the system and therefore decreased the overall research productivity. Both policies combined consistently outperformed isolated policies. Figure 3 is representative of most simulations, being robust under different assumptions for the sensitivity analysis.

## Discussion

To our knowledge, this is the first article describing an open source application for collaborative sharing of metadata focusing on epidemiological and biomedical research. We have then described a policy model putting this application in a broader research policy context to demonstrate that an isolated metadata sharing policy is superior to an isolated data-sharing policy, and that both policies combined achieve the best results in terms of publications, thus emphasizing the important role of the DoD in this context.

The DoD describes study as well as variable level description related to a biomedical research database. The former enables cataloging that in turn facilitates search across databases while the latter imparts granularity to the search mechanism. The DoD presents opportunities for both database owners as well as other researchers prospecting for an existing database. It serves as a sort of marketing mechanism by which a database owner can reuse and achieve optimal utilization of his/her own data. Additional benefits include opportunities to collaborate, apply for joint funding, joint publication. In the absence of this mechanism, a database is frequently underutilized of its true potential on account of paucity of time, research questions, funding and expertise. On the other hand, prospective researchers looking for a database matching their research question/idea can do a variable as well as a categorical search. They can then evaluate the resultant list of databases and variables to see what suits them best thus saving time, effort and cost involved in doing a prospective study.

Since, data sharing is not a prevalent and preferred trend among database owners, metadata sharing through a web based application is a valid alternative. The receptivity is enhanced as part of information shared under this mechanism is already in the public domain, in the form of database description in methods section of peer reviewed publications. In this way, concerns about confidentiality are addressed. Further since this application provides due recognition and acknowledgment to the database owner and does not share actual data, concerns related to propriety and security are nullified. The DoD is also equipped with a wiki mechanism which enables data provenance. End-user researchers can check the changes in the database as well as its variables. This information is critical while conducting a secondary data analysis with existing data. Additionally end -user researchers can add their observations in terms of the data quality based on their experience with analyzing the database. The application also encourages sharing of research questions in the study description page. Busy database owners can list potential research questions which other researchers can explore in collaboration with them, again ensuring optimal utilization of existing data and discouraging data kept in silos. Finally, listing citations of peer-reviewed publications that were based on the dataset validates it and serves as a dynamic guide about its data quality.

Previous efforts to share biomedical research data include Biomedical Informatics Research Network (BIRN), caBIG [4], among many others [21]–[26]. Development of caBIG is a revolutionary step in the field of cancer research, not only in terms of data sharing but also in terms of achieving data standards using common data elements (CDEs) for cancer research. However, these efforts are currently limited to few areas including cancer, cardiology, tuberculosis, and a few others, their extension to several other disciplines is needed. The effort of making an interdisciplinary database is a challenging task but necessary given the overlapping nature of healthcare. Additionally when it comes to interdisciplinary biomedical research, the task of defining CDEs becomes challenging. We believe that a well-populated metadata repository like DoD can serve as examples that can guide CDEs in multiple biomedical research areas. Similar and repeated variables in DoD can then assist in the development of common data elements in a biomedical research field/domain.

Another caBIG sponsored initiative related to our project is caTRIP (Cancer Translational Research Informatics Platform), a tool aiming to increase the network of researchers by integrating clinical and molecular data in a repository. caTRIP extends beyond outcomes analysis and research to actual patient care by allowing clinicians to get focused search results to meet the individual patient needs based on the available information [27]. Although caTRIP is a promising project, adherence regarding researchers using the application to deposit data is still in its initial stages. According to our model, a mechanism that allows for meta-data sharing would therefore contribute to an environment where data are shared. Also, given the concerns related to ownership of data, competitive edge and data security issues, we believe DoD can complement caTRIP in its effort of creating a robust and widely acceptable data sharing platform for cancer researchers. As DoD encourages sharing of metadata and not the actual patient data, several of the concerns outlined earlier are diminished.

Other efforts have focused exclusively on information on databases rather than study data sets, such as a recent repository created by NHLBI (National Heart, Lung and Blood Institute) (http://www.nhlbi.nih.gov/resources/deca/directry.htm) and other previous systems [28]–[31]. These efforts have received varying levels of success, especially in small domains and were limited by the social culture of biomedical research. Despite the multiple benefits of a metadata repository, few efforts have been directed at utilizing this method to complement data sharing in biomedical research. Notably, Niland [32] included a metadata repository in the informatics blueprint for healthcare quality information systems (HQIS) as it enables; 1. effective retrieval, utilization and management of data 2. Accurate analysis and reporting of HQIS data. There are instances where efforts have been directed at building metadata repositories 1. as a part of healthcare data management to enhance data quality [33] and 2. to support research through integration of data systems [34]. The latter effort implemented at City of hope national medical centre, aimed to manage data in a research data warehouse and facilitate complex, multi disciplinary analyses [34]. The DHS Department of Human Services of Australia, built a metadata repository to support the data collection reform process with DHS Metadata Repository (http://www.health.vic.gov.au/hacims/reforms/metadata.htm). Ministry of Health, British Columbia and IBM went a step further by building a ‘repository of repositories’ equipped with features such as 1. web based interface 2. distributed metadata administration, 3. configurable security and 4. annotation capability [35]. These efforts were pursued from the data management perspective and possess limited ability and features to support biomedical research. To our knowledge, the DoD is the first open source application while emphasizing a collaborative environment. By facilitating the modification of information on study databases as well as research questions, the system has the potential to increase information access and opportunities for collaboration, thus accelerating the advance of medical science in general.

It is common belief among funding and governmental agencies that data sharing should be the main policy for making the best use of biomedical research data, ultimately contributing to the improvement of healthcare. Despite this belief, resistance from researchers and epidemiologists themselves is frequent, researchers arguing that making their data freely available will lower their competitive advantage in an environment where researchers fight for funding. The results of our policy model point in a different direction. While it is acknowledged that a data-sharing policy would certainly improve the current status of clinical and epidemiological research, our model also pointed to the unintended consequence of data being shared with poor quality. In contrast, policies emphasizing the sharing of metadata significantly improved overall data sharing, while also being more amenable to researchers and epidemiologists since their “ownership” is not violated. Although this model is based on a focus group and has not been validated against quantitative data that would further validate its results, our model points to an interesting alternative that deserves consideration by research policy makers.

Although, the DoD and our policy model are significant advances in relation to the previous literature, our study has limitations. First, at this point the DoD is still being spread in terms of its user base, and its impact on research collaborations still needs to be validated in future studies. In relation to our policy model, the validation was performed using expert opinion from a small group of epidemiologists and clinical researchers currently using the DoD, but this pool should be expanded in future validations.

Based on the results of our study, the use of information about databases should be further explored as a mechanism to expand collaboration among biomedical researchers. Future studies should focus on the integration with larger social network systems through open platforms such as OpenSocial (http://code.google.com/apis/opensocial/), thus substantially expanding the reach of the collaboration network. In addition, scalable technologies for acquisition of information about research resources such as text mining from full-text articles should be evaluated in association with social networks. Finally, policy models should take into account these new technologies, specially evaluating how they will affect different generations of researchers with a wide range of comfort levels in relation to computer use and social networks.

## Supporting Information

Figure S1 Advanced search interface and search results.
(0.98 MB TIF)

Figure S2 Search results in DoD.
(0.99 MB TIF)

Figure S3 Database general information.
(0.63 MB TIF)

Figure S4 List of variable names in a data dictionary.
(0.93 MB TIF)

Figure S5 Adding Database information.
(0.46 MB TIF)

Appendix S1 (0.03 MB DOC)
