# Supplementary figures and images for: Application Description and Policy Model in Collaborative Environment for Sharing of Information on Epidemiological and Clinical Research Data Sets
Source: PLoS One. 2010 Feb 19;5(2):e9314. doi: 10.1371/journal.pone.0009314 (PMC2824801; doi:10.1371/journal.pone.0009314)

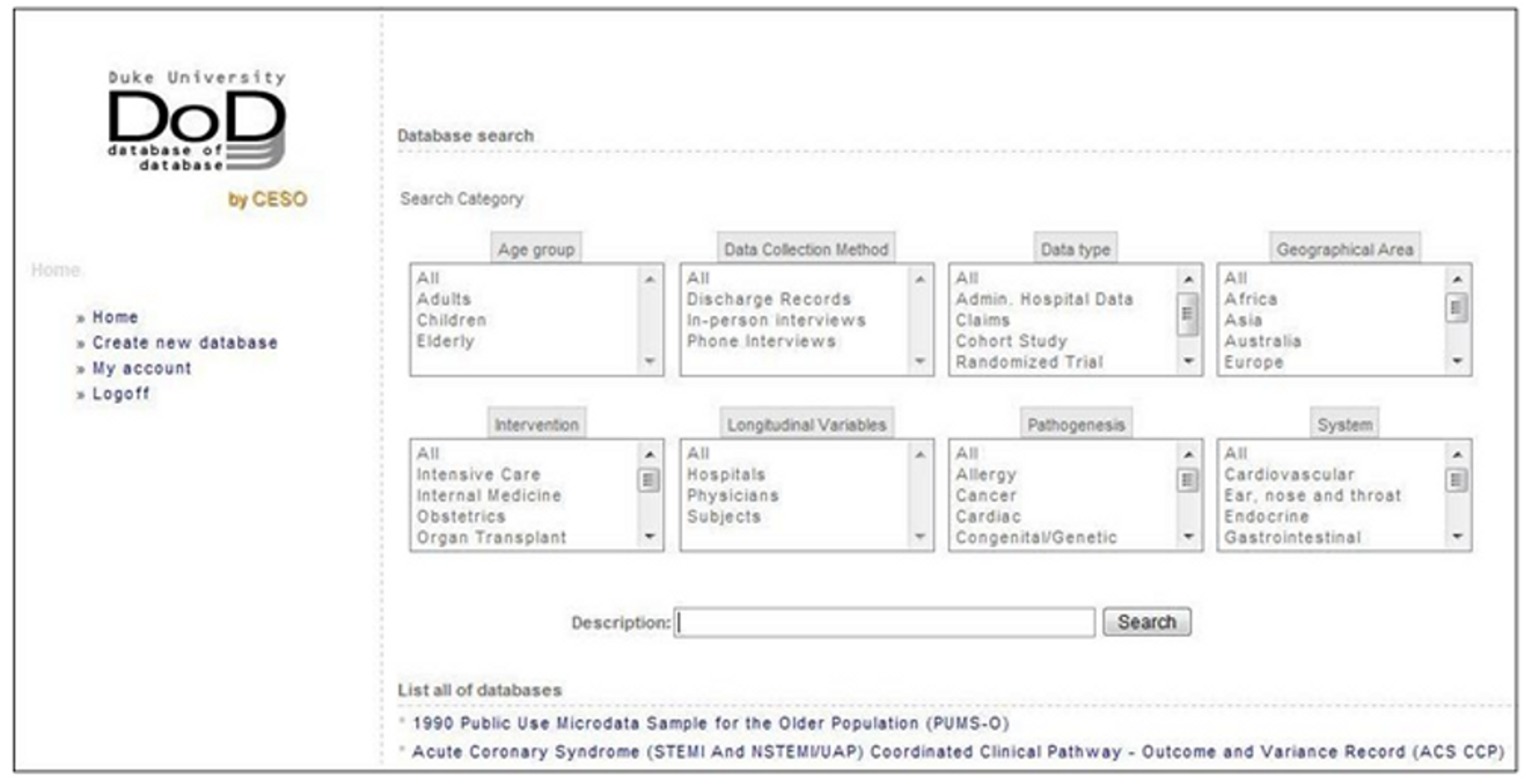

Supplement: Figure S1 — Advanced search interface and search results. (0.98 MB TIF) [file pone.0009314.s001.tif]

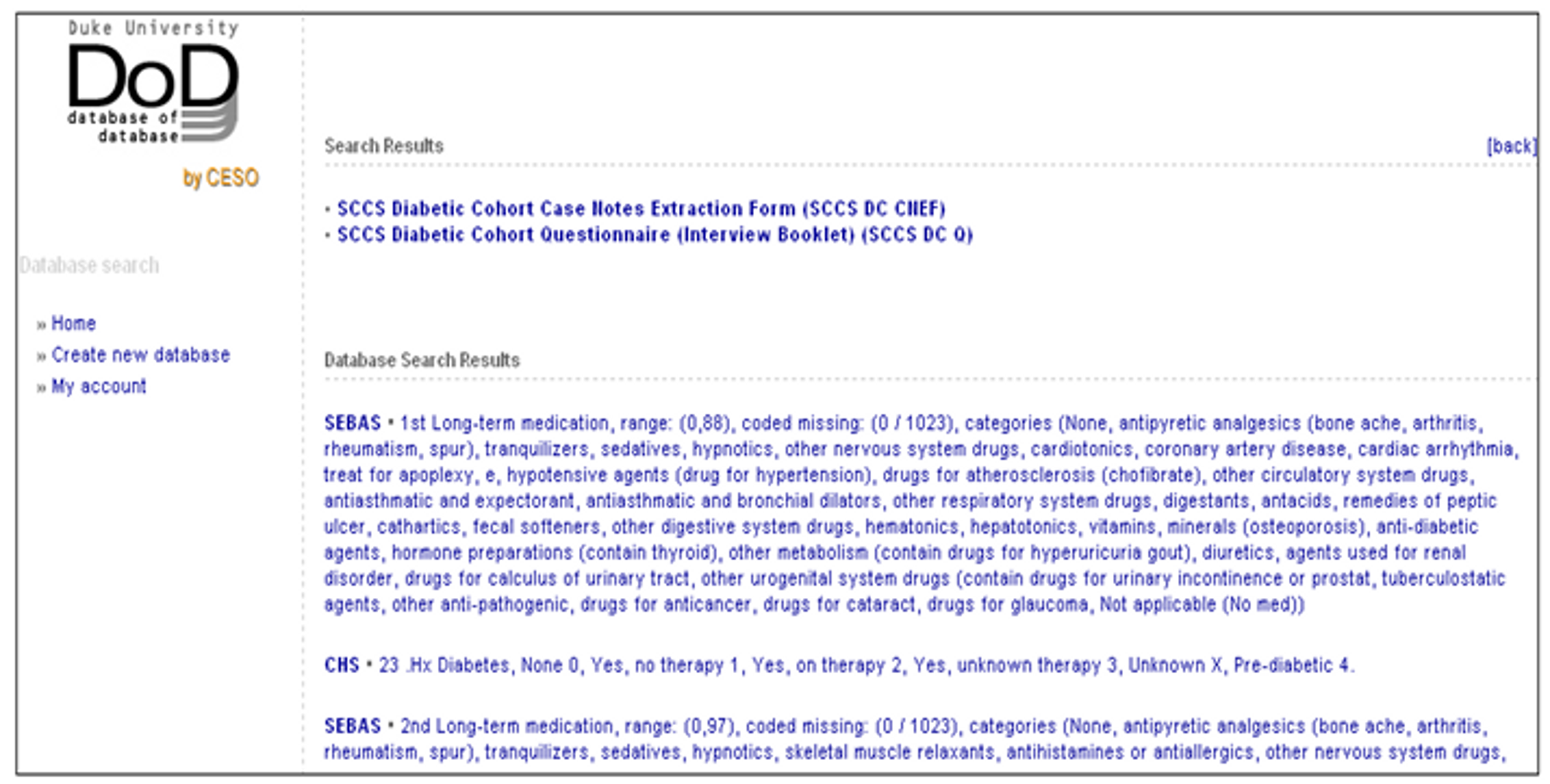

Supplement: Figure S2 — Search results in DoD. (0.99 MB TIF) [file pone.0009314.s002.tif]

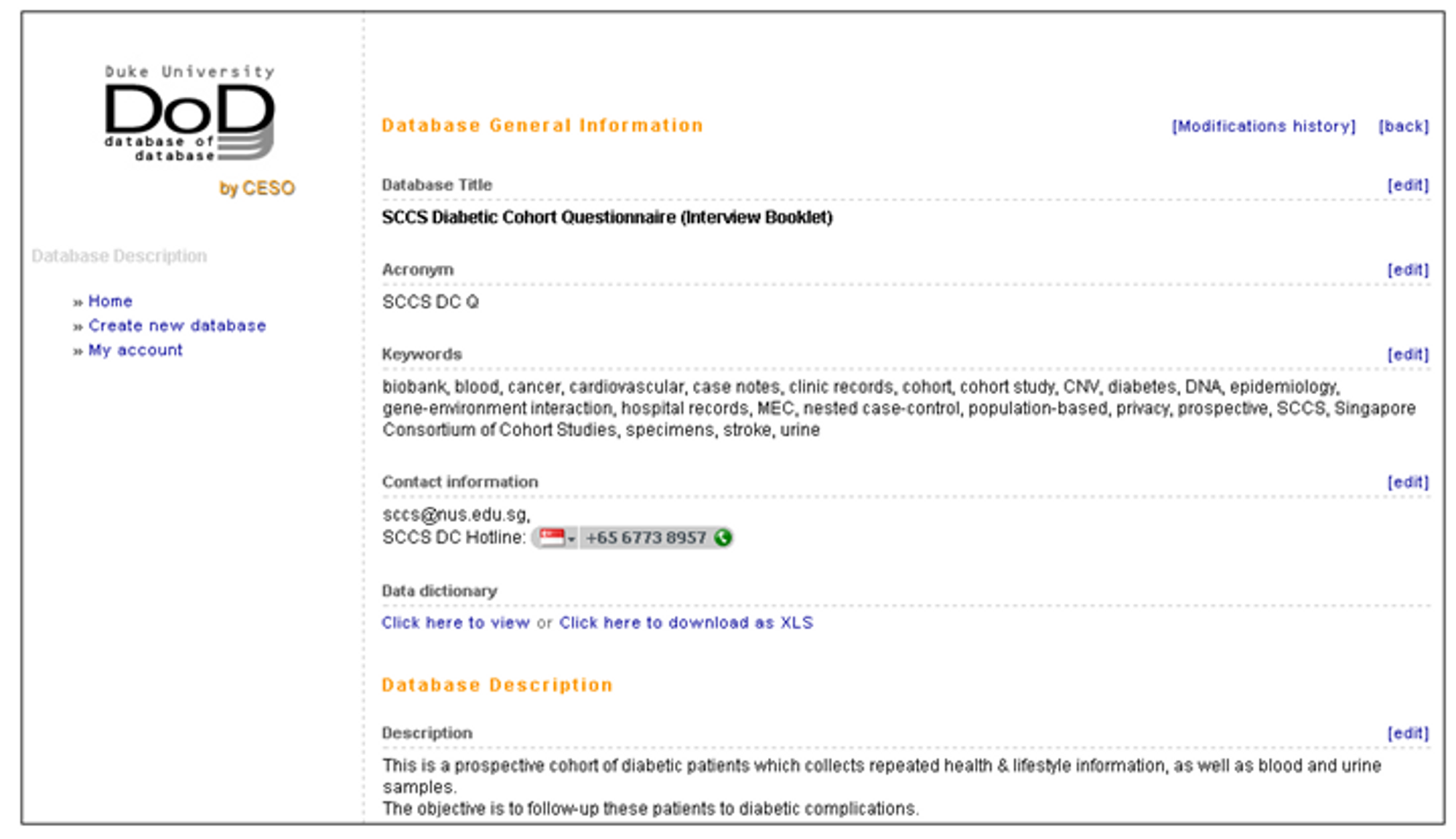

Supplement: Figure S3 — Database general information. (0.63 MB TIF) [file pone.0009314.s003.tif]

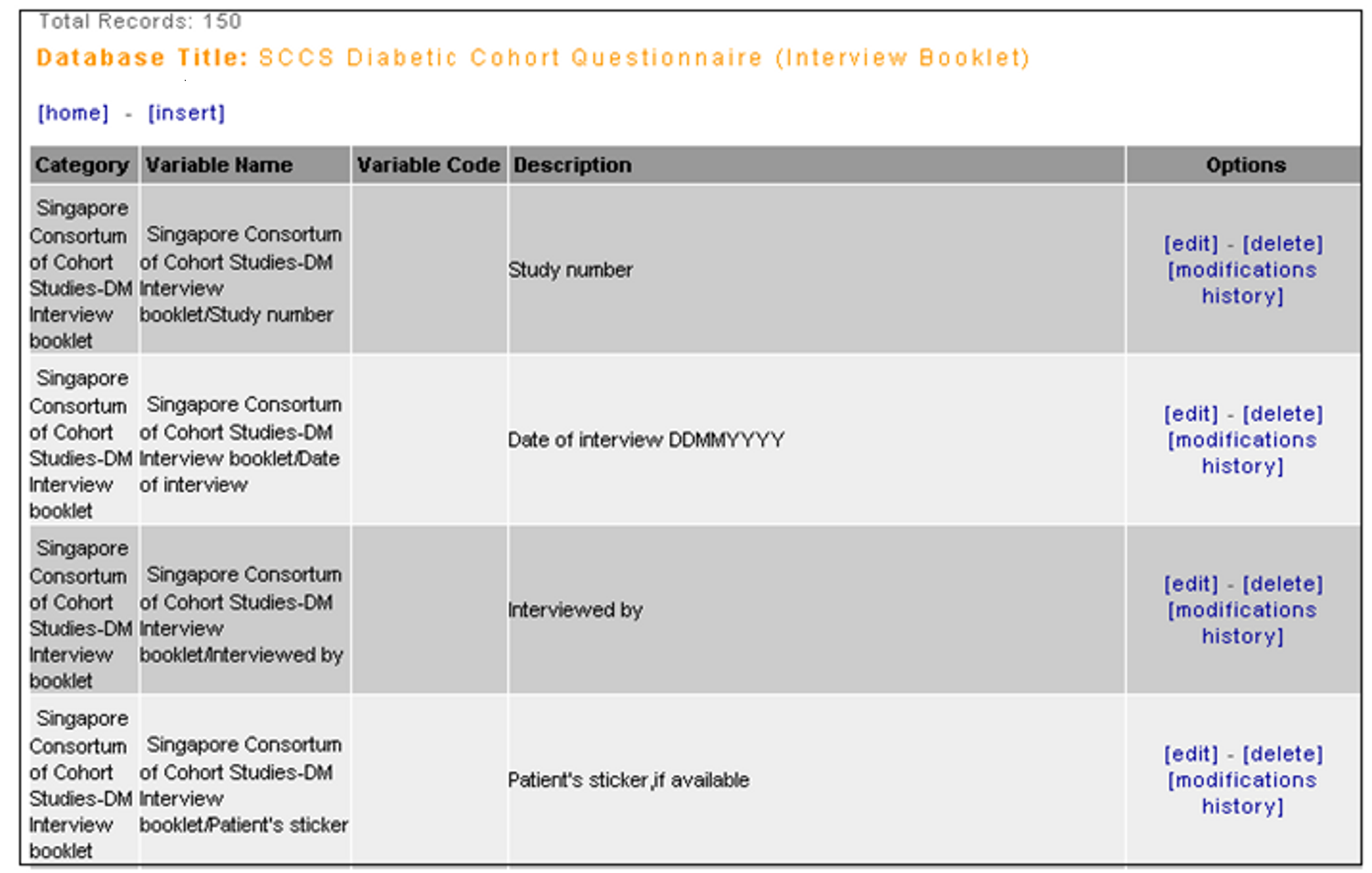

Supplement: Figure S4 — List of variable names in a data dictionary. (0.93 MB TIF) [file pone.0009314.s004.tif]

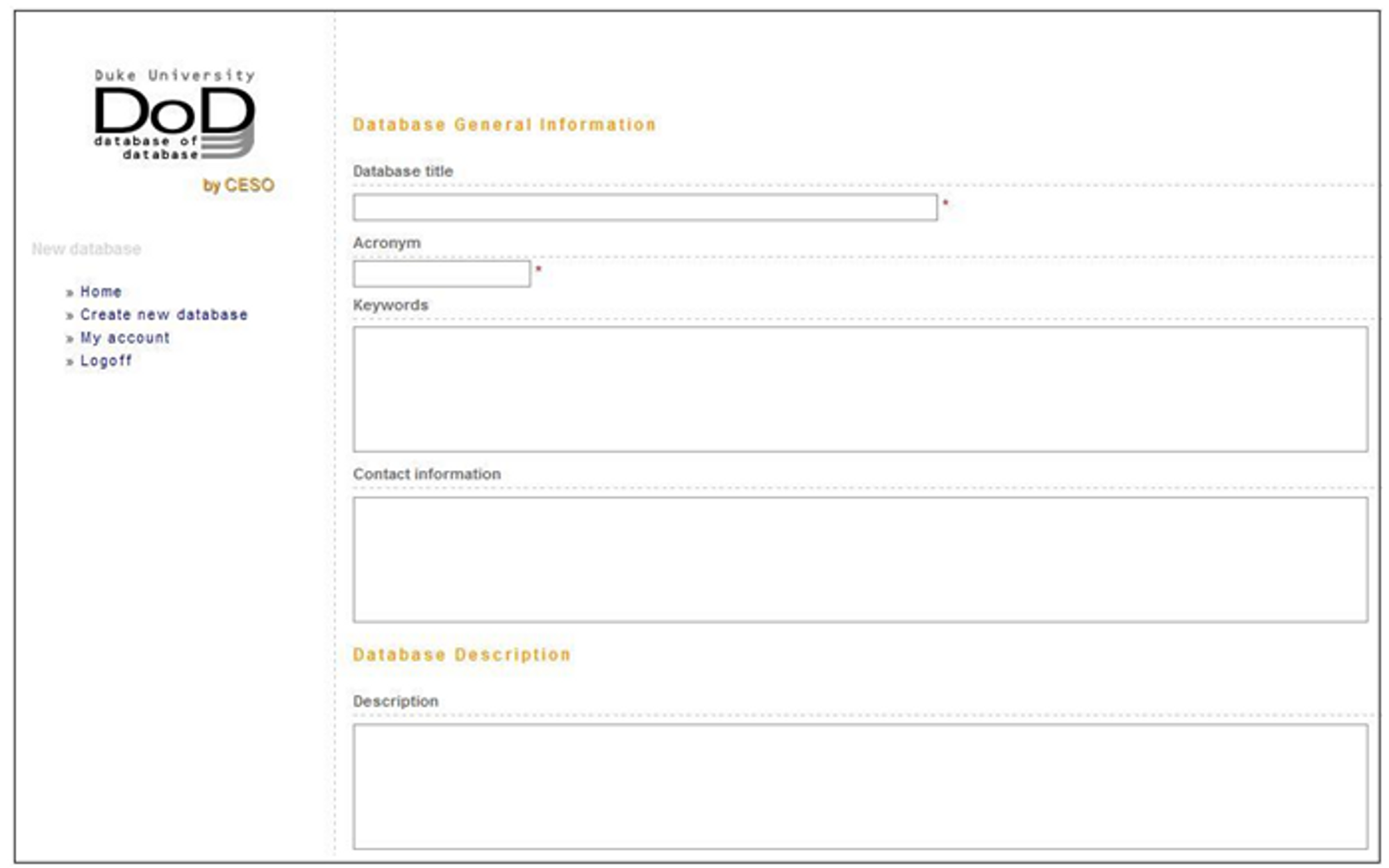

Supplement: Figure S5 — Adding Database information. (0.46 MB TIF) [file pone.0009314.s005.tif]
