# Supplementary material for: Application Description and Policy Model in Collaborative Environment for Sharing of Information on Epidemiological and Clinical Research Data Sets
Source: PLoS One. 2010 Feb 19;5(2):e9314. doi: 10.1371/journal.pone.0009314 (PMC2824801; doi:10.1371/journal.pone.0009314)
Supplement: Appendix S1 — (0.03 MB DOC) [file pone.0009314.s006.doc]

## APPENDIX S1

## Software Architecture and requirements

DoD was modeled under a MVC (Model–View–Controller) paradigm [23], and programmed using the PHP language (Hypertext Preprocessor, <http://php.net/>), its database was implemented in MySQL [26], and released under the GNU General Public License (GPL, <http://www.gnu.org/copyleft/gpl.html>).  A free download of the source code can be made from <http://sourceforge.net/projects/databaseofdatab/>. Minimum hardware requirements for installation are a Pentium IV processor with 15 MB of free hard drive space and 512 MB of RAM. The software requires Apache [27], PHP [25], and MySQL [26], and can be installed on Windows or Linux operating systems.  The current application operates from a central server located at Duke University, with a mirror in Asia to increase access speed.

## Operation

On the home page, a basic search field enables keyword searches through the information of all study databases.  An additional Advanced Search option (Figure S1) is also available with pre-set search parameters.

Search results are displayed as a set of links in two separate lists (Figure S2).  The first list, "Search Results," consists of results where the keyword is found on the database title. The second list, "Database Search Results," contains variable names having the searched keyword.  Variable names are listed along with acronyms for their respective databases.

Clicking on the name of a database displays the Database General Information page (Figure S3), while selecting "Click here to view" under "Data dictionary" returns a list of all variable names in that database (Figure S4).

The application also allows researchers to create an account so that they can publish information about their own study databases (Figure S5).

## Figures

Figure S1 - Advanced search interface and search results

Figure S2 - Search results in DoD

Figure S3 - Database general information

Figure S4 - List of variable names in a data dictionary

Figure S5 – Adding Database information
